# Eosinophils in filarial infections: Inducers of protection or pathology?

**DOI:** 10.3389/fimmu.2022.983812

**Published:** 2022-10-31

**Authors:** Alexandra Ehrens, Achim Hoerauf, Marc P. Hübner

**Affiliations:** ^1^ Institute for Medical Microbiology, Immunology and Parasitology, University Hospital Bonn, Bonn, Germany; ^2^ German Center for Infection Research (DZIF), Partner Site Bonn-Cologne, Bonn, Germany

**Keywords:** filaria, eosinophil, lymphatic filariasis, onchocerciasis, granulocyte, ETosis, EPO, TPE

## Abstract

Filariae are parasitic roundworms, which can cause debilitating diseases such as lymphatic filariasis and onchocerciasis. Lymphatic filariasis, also known as elephantiasis, and onchocerciasis, commonly referred to as river blindness, can lead to stigmatizing pathologies and present a socio-economic burden for affected people and their endemic countries. Filariae typically induce a type 2 immune response, which is characterized by cytokines, i.e., IL-4, IL-5 and IL-13 as well as type 2 immune cells including alternatively activated macrophages, innate lymphoid cells and Th2 cells. However, the hallmark characteristic of filarial infections is a profound eosinophilia. Eosinophils are innate immune cells and pivotal in controlling helminth infections in general and filarial infections in particular. By modulating the function of other leukocytes, eosinophils support and drive type 2 immune responses. Moreover, as primary effector cells, eosinophils can directly attack filariae through the release of granules containing toxic cationic proteins with or without extracellular DNA traps. At the same time, eosinophils can be a driving force for filarial pathology as observed during tropical pulmonary eosinophilia in lymphatic filariasis, in dermatitis in onchocerciasis patients as well as adverse events after treatment of onchocerciasis patients with diethylcarbamazine. This review summarizes the latest findings of the importance of eosinophil effector functions including the role of eosinophil-derived proteins in controlling filarial infections and their impact on filarial pathology analyzing both human and experimental animal studies.

## 1 Filariae

**Figure 1:**
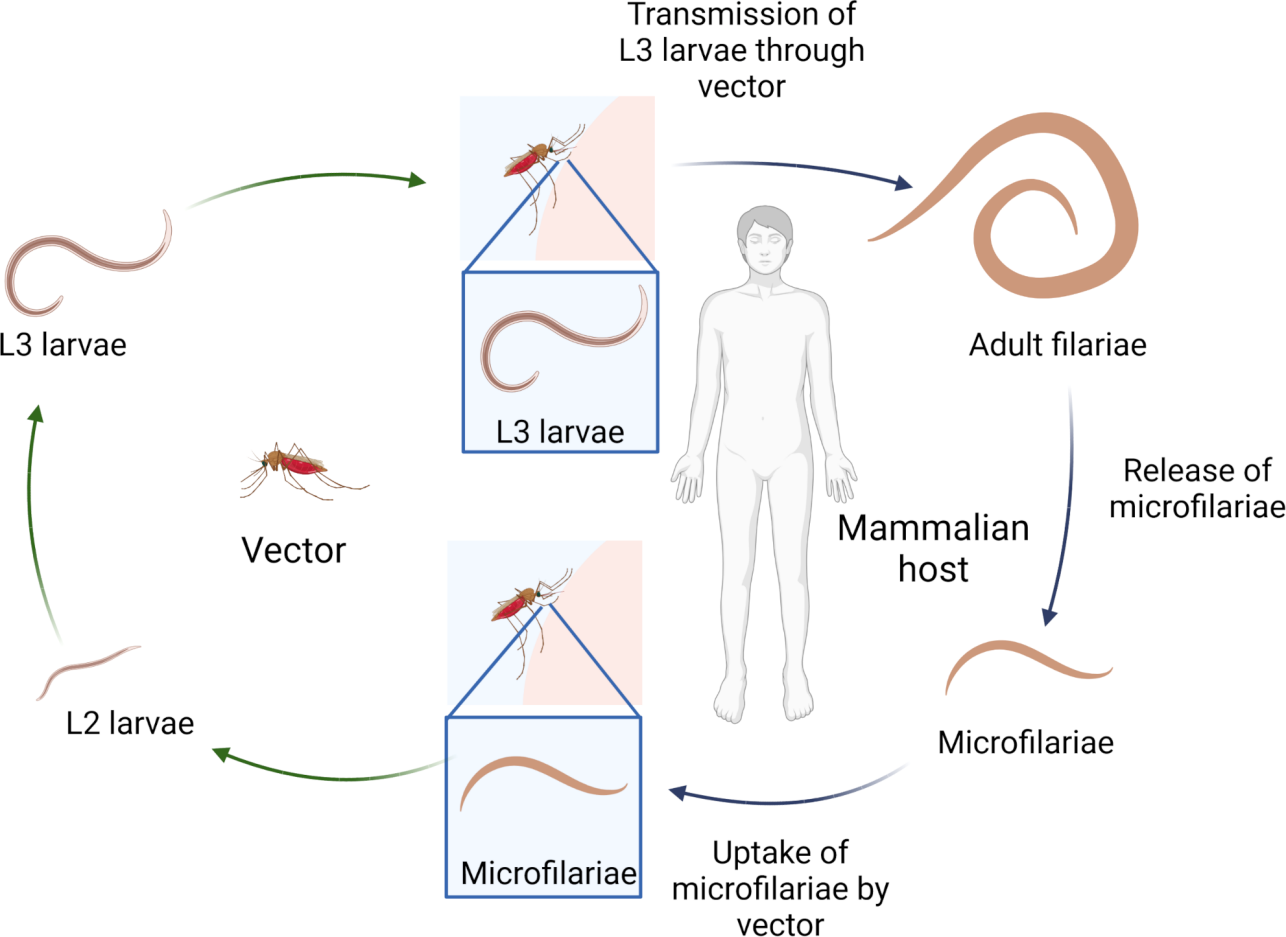
Filarial life-cycle. Created with BioRender.com.

Filariasis are vector-borne diseases caused by filarial nematodes. Infection occurs *via* blood-feeding insects (e.g. mosquitoes) that transmit the infective third stage larvae (L3) during their blood meal. Following two moltings, adult filariae develop in the definite mammalian host, mate and start to release their progeny, the microfilariae (MF). Dependent on the filarial species, MF are mainly found in the peripheral blood or skin. Upon ingestion of the MF (L1 stage) during another blood meal of the appropriate insect vector, MF develop *via* two moltings into the L3 stage to complete the life-cycle (**Figure 1**) (1). Filariae cause several tropical diseases including onchocerciasis, lymphatic filariasis (LF), loiasis and mansonellosis. While some filariae, such as the pathogenic agents for onchocerciasis and LF, can cause severe pathology in infected patients, others like the filariae inducing loiasis and mansonellosis, lead mainly to asymptomatic infections and only occasionally cause clinical symptoms (2–4). A summary of the causative agents, the geographical distribution and estimated number of infected individuals, the associated clinical symptoms and the vectors transmitting the diseases is given in **Table 1**. In short, onchocerciasis, is caused by *Onchocerca volvulus*, and an estimated 21 million people are infected, which can lead to vision impairment or blindness (1.15 million people) as well as severe dermatitis (15 million people) (1, 5). LF is caused by *Brugia malayi*, *Brugia timori* and *Wuchereria bancrofti* with 51 million people infected. 17 million LF patients suffer from lymphedema in the lower extremities and 20 million men from lymphedema in the scrotum (hydrocele). Few LF patients develop tropical pulmonary eosinophilia (TPE), which is characterized by asthma-like symptoms (1, 6). Loiasis, the eye-worm, is caused by *Loa loa*, which can lead to edema (calabar swellings) and temporary painful sensations in the eye, which are both a results of the migrating adult filariae (4). Mansonellosis is caused by *Mansonella perstans*, *Mansonella ozzardi* and *Mansonella streptocerca. M. perstans* is most common with an estimated 120 million infections, although it represents one of the most neglected filarial diseases, as there are no specific symptoms associated, but unspecific symptoms such as abdominal pain, headache and subcutaneous swellings are repeatedly reported (7) (**Table 1**). Importantly, following treatment with MF-killing (microfilaridical) drugs, most severe adverse events can occur, such as life-threatening encephalitis in loiasis patients or severe dermatitis and blindness in onchocerciasis patients treated with diethylcarbamazine (DEC) (14, 15).

**Table 1:** Filariae and human filarial diseases.

Filarial disease	Causative agent	Vector	Worm location	Disease symptoms	Cause of symptom	Number of cases	Distribution	Ref.
**Human filariae**								
**Onchocerciasis (River blindness)**	*Onchocerca volvulus*	Blackflies (*Simulium* species)	Adults: Subcutaneous nodules (onchocercomata) MF**:** Skin and subcutaneous tissue	Eye: Vision impairment, blindness Skin: Dermatitis, depigmentation, papules, hanging groin	Immune response towards MF	21 million infected 15 million with skin manifestations 1.15 million with visual impairment	99% of infected people live in sub-Saharan, Africa Remaining foci in Brazil and Yemen	(1, 5, 8–10)
**Lymphatic filariasis (Elephantiasis)**	*Wuchereria bancrofti, Brugia malayi*, *Brugia timori*	Mosquito species *Mansonia*, *Aedes*, *Anopheles* and *Culex*	Adults**:** Lymphatics MF: Blood and lymphatics	Lymphedema in lower extremities and scrotum (hydrocele) Occasional tropical pulmonary eosinophilia ((TPE): asthma-like symptoms)	Immune response towards, adults TPE: trapping of MF in the lung	51 million infected people 20 million with hydrocele 17 million with lymphedema	Mainly found in sub-Saharan Africa, South-East Asia, India, and Latin America	(1, 5, 6, 11)
**Loiasis (African eye-worm)**	*Loa loa*	Chrysops flies	Adults: Subcutaneous tissue (eye and skin) MF: Blood	Mainly asymptomatic Occasional local angioedema (Calabar swelling), eye worm, urticaria, pruritus, endomyocadial fibrosis	Calabar swelling and eye-worm: migration of, adults Endomyocardial fibrosis: hyperosinophilia, Severe adverse effects to DEC		Central and Western Africa	(4, 12, 13)
**Mansonellosis**	*Mansonella perstans*, *Mansonella ozzardi*, *Mansonella streptocerca*	Midges of the genus *Culicoides*	Adults: Peritoneum or pleura MF: Blood, skin (only *M. streptocerca*)	Mainly asymptomatic Occasional subcutaneous swellings, pericarditis and pleuritic, ocular symptoms, abdominal pain, headache	Migration of adult worms, Ocular symptoms: MF	100 million people	Western, Eastern and Central Africa, parts of Central and South America, Caribbean islands	(3, 7)

Causative agents, vectors, symptoms and epidemiology of filarial diseases.

To study filariae, animal models have been extensively used and contributed to our current understanding about the protective immune responses, filarial immunomodulation and allowed to identify new drugs against filariae (16–18). Given that mice are not susceptible to human-pathogenic filariae, surrogate filarial nematode species are often used. *Onchocerca ochengi* is a natural parasite of cattle and is closely related to the human filarial nematode *O. volvulus*. Similarly to the human infection, adult worms reside in subcutaneous nodules, while MF can be found in the skin (**Table 2**) (21). It is regularly used to test antifilarial drugs and vaccines (22–24, 33, 34). Gerbils and immunodeficient mice also maintain *O. ochengi* or *B. malayi* filariae after intraperitoneal implantation, which allows preclinical testing of drug candidates in a small rodent model (35–37). To study LF, the *B. malayi* ferret or mouse model can be used, where *B. malayi* L3 are injected into the footpath of ferrets or hind limb of mice, leading to adult worm development in the lymphatics and lymphedema in the limb (**Table 2** **)** (27, 28, 38). Onchocerciasis-induced keratitis on the other hand is investigated in mice injected with *O. volvulus* antigen or MF, which leads to opacification and keratitis (25, 26, 39). To study the immune response to filariae in immunocompetent mice, the filarial *Litomosoides sigmodontis* model is an excellent tool, since the induced immune responses resemble those observed in human filarial infections (**Table 2** **)** (40, 41). *L. sigmodontis* L3 larvae are transmitted through the bite of the tropical rat mite *Ornithonyssus bacoti* during a blood meal; the L3 larvae migrate through the skin *via* the lymphatics to the pleural cavity. Gravid females start to release MF into the peripheral blood, which can be taken up again by the mites. Within the mites, the MF develop into the L3 stage (19).

**Table 2:** Animal models to study filariasis.

Filarial disease model	Causative agent	Vector	Worm location	Disease symptoms	Ref.
**Animal model**					
**Onchocerciasis, LF**	*Litomosoides sigmodontis* in cotton rats, mice and mongolian gerbils	Mites (*Ornithonyssus bacoti*)	Adults: Pleura and occasionally, peritoneum MF: Blood	Not applicable	(19, 20)
**Onchocerciasis in general**	*Onchocerca ochengi* in cattle	Blackflies (*Simulium* species)	Adults: Subcutaneous nodules (onchocercomata) MF**:** Skin and subcutaneous tissue	Not applicable	(21–24)
**Onchocerciasis keratitis**	*Onchocerca volvulus* antigen or MF, *Wolbachia*-containing extract injected into the eye of mice after immunization	Not applicable	Not applicable	Ocular lesions in the eye	(25, 26)
**LF lymphedema**	*Brugia malayi* in ferrets	Subcutaneous injection of L3 larvae	Adults**:** Lymphatics in lower extremities and, genitals MF: Blood and lymphatics	Lymphedema in food path	(27, 28)
**LF/onchocerciasis cell migration**	*B. malayi * or *O. volvulus* in mice	Peritoneal injection/transplantation of L3 larvae in diffusion chamber	Adults**:** Peritoneum MF: -	Not applicable	(29–31)
**LF TPE**	*L. sigmodontis or B. malayi* MF sensitization and challenge in mice	Not applicable	Adults: - MF: Blood, lung and bronchoalveolar fluid	Lung pathology	(32)

Studying model organisms has advanced our knowledge on protective immunity and pathology development during filarial infections. Thus, it was demonstrated that filarial infections provoke a type 2 immune response in the host with eosinophilia being a hallmark of filarial infections. Eosinophils are the predominant cell type during filarial infection and they contribute to protection and the development of pathogenesis. Several reviews published so far, described the role of eosinophils and other granulocytes during helminth infections in general (42–44). However, they focused almost exclusively on intestinal helminths and blood flukes, while the present review summarizes the current state-of-the-art knowledge on eosinophil-mediated protection and pathology during filarial infections.

## 2 Eosinophils and their effector functions

### 2.1 Eosinophil development and activation

Filarial infections provoke a type 2 immune response in the host, which is initiated by epithelial cell-derived alarmins such as thymic stromal lymphopoietin (TSLP), Interleukin (IL)-25 and IL-33 as a response to the tissue damage caused by the multicellular parasites (45). In response, type 2-related cytokines including IL-4, IL-5, IL-9, IL-10, and IL-13 are produced (46, 47), which support the induction and expansion of innate lymphoid type 2 cells (ILC2s), eosinophils, alternatively activated macrophages (AAMs) and T helper 2 cells, as well as antibody isotypes IgG1 (mouse), IgG4 (human) and IgE (48). A hallmark of filarial infections is a significant increase of blood eosinophils (from 120/mm^3^ under homeostatic conditions to >450/mm^3^) (49, 50).

Under homeostatic conditions, eosinophils are derived from the bone marrow and migrate quickly into the tissue, primarily the gastrointestinal tract, the lung, the uterus, mammary gland tissue, as well as adipose tissue and thymus (51–54). During allergy and filarial infection eosinophil numbers significantly increase and eosinophils are recruited in high numbers to the sites of tissue repair and inflammation (53, 55). While eosinophils have been shown to be involved during bacterial and viral infections, eosinophils contribute to control helminth infections as well (51). Tissue damage caused by the migrating filariae triggers the production of the alarmin IL-33, mainly by dying epithelial and endothelial cells, adipocytes and fibroblasts (51, 56). Furthermore, the alarmin IL-25 is produced by Th2 cells, mast cells and eosinophils. Both alarmins induce IL-5 release by Th2 cells and ILC2s. IL-5, together with IL-3 and granulocyte-macrophage colony-stimulating factor (GM-CSF), drives the development of eosinophils in the bone marrow. Moreover, IL-5 is a key cytokine not only involved in the development of eosinophils but also in the priming and activation of eosinophils (57). IL-4 and IL-13, produced by Th2 cells and ILC2s, impact eosinophil recruitment as well. Both cytokines signal *via* the IL-4R, which is expressed among others on fibroblasts and epithelial cells, leading to the release of chemokines, which stimulate eosinophil migration. These chemokines include the eotaxins CCL11 (eotaxin 1), CCL24 (eotaxin 2) and CCL5 (RANTES) (58). Other sources of eotaxins include eosinophils, monocytes, lymphocytes, dermal fibroblasts, epithelial cells and macrophages (59–62). IL-4 and IL-13 also induce the upregulation of the adhesion molecule VCAM-1 on endothelia cells at the site of infection and thus enhance the adhesion of eosinophils and their local accumulation (57, 63, 64).

### 2.2 Eosinophil effector functions

Eosinophils are equipped with Fc receptors as well as pattern-recognition-receptors (PRR), which enables them to recognize pathogen-associated molecular patterns (PAMPs) and damage-associated molecular patterns (DAMPs) (51). As response to PAMPs and DAMPs, eosinophils can interact with other cells either through the expression of MHCII molecules, by releasing cytokines and chemokines or by mediating the release of their intracellular granules containing toxic proteins (51). Thus, eosinophils can interact with T cells through MHCII expression and drive their proliferation and cytokine production; they can also mediate Th2 cell recruitment through release of the chemoattractant molecules CCL22 and CCL17 (65, 66). Moreover, eosinophils drive the maturation of AAMs through the release of IL-4 and IL-13, supporting tissue repair through fibroblast recruitment and tissue remodeling (67).

A pivotal eosinophil effector mechanism is the release of their cytotoxic granules, which contain anti-microbial peptides and inflammatory mediators that support the elimination of invading pathogens and enhance the ongoing inflammation. Eosinophils store different types of secretory organelles in the cytosol including the most abundant form, the crystalloid granules, as well as primary granules, small granules and secretory vesicles. The crystalloid granules mainly contain four highly basic proteins, namely major basic protein (MBP), eosinophil cationic protein (ECP), eosinophil-derived neurotoxin (EDN) and eosinophil peroxidase (EPO), while the primary granules contain Charcot-Leyden crystal (CLC) forming proteins (51). MBP is the most highly cationic protein in eosinophil granules (68). Furthermore, the granules contain two ribonucleases A: EDN, which shows strong antiviral activity, and ECP, which has been described to form pores rather than have ribonuclease activity (69). Equivalent to the myeloid peroxidase found in neutrophils, EPO is a haloperoxidase and is associated with bacterial killing through the production of reactive oxygen species (ROS) (53). Lastly, CLC-forming proteins can be found in primary granules of human, but not murine eosinophils (70). CLC are formed upon the release of galectin-10 proteins, which accumulate and form hexagonal and bipyramidal-shaped crystals (71–73) (**Box 1**). CLC were described for tape worm infections, filarial diseases and other eosinophil-associated diseases such as allergies and asthma (71, 72, 80, 81).

Box 1 **Eosinophil granule proteins**

Crystalloid granules
Major basic protein (MBP)
• Reduces worm burden in *L. sigmodontis*-infected mice (74)
• Is deposited on *O. volvulus* MF during Mazzotti reaction (75)
• Deposit on *B. malayi* MF in TPE (76)
• Kills *B. malayi* and *B. pahangi* MF *in vitro* (77)
Eosinophil cationic protein (ECP)
• Deposit on *O. volvulus* MF in lymph node after diethylcarbamazine (DEC) treatment (78)
• Kills *B. malayi* and *B. pahangi* MF *in vitro* (77)
Eosinophil-derived neurotoxin (EDN)
• Kills *B. malayi* and *B. pahangi* MF *in vitro* (77)
Eosinophil peroxidase (EPO)
• Reduces worm burden in *L. sigmodontis*-infected mice (74)
• Is deposited on *O. volvulus* MF in lymph node after DEC treatment (78)
• Kills *B. malayi* and *B. pahangi* MF *in vitro* in combination with ROS (77)

Primary granules
Charcot-Leyden crystal (CLC) proteins
• Found in tape worm infections and filarial diseases (71)
• Contributes to eosinophil ETosis (79)

In general, the release of eosinophil granules can be mediated through several mechanisms. Following crosslinking of Fc receptors such as FcγRII, FcγRIII, FcαRI and FcϵRI by IgG1, IgG3, IgG2, IgA and IgE, antibody-dependent cellular cytotoxicity (ADCC) occurs, leading to cell degranulation, activation and/or phagocytosis. Especially during secondary filarial infections and after vaccination, eosinophil-mediated ADCC plays an important role in the killing of the filariae (29, 82, 83). Furthermore, PAMPs and DAMPs can be recognized through PRR expressed on eosinophils such as toll-like receptors (TLRs) (TLR1, 2, 3, 4, 5, 6, 7, 9 and 10) or C-type lectin receptors (CLR) (51). TLR2 and TLR6 are of particular importance, as filariae including the causative agents of LF, onchocerciasis and mansonellosis, but not loiasis, contain endosymbiotic *Wolbachia* bacteria, which trigger an inflammatory response through TLR2 and TLR6 recognition (8, 84). *Wolbachia* can also be recognized by the nucleotide-binding oligomerization domain-containing protein 2 (NOD2), which supports neutrophil recruitment to the skin and subsequent L3 larval elimination in murine filariasis (85). The binding of TLR and CLR ligands leads to the release of granules containing cytotoxic cationic proteins as well as cytokines and chemokines through exocytosis (granules fuse with the plasma membrane), piecemeal degranulation (shuttling of the granular content *via* secretory vesicles from the granules to the plasma membrane) and cytolysis (rupture of the plasma membrane). The latter can be associated with or without extracellular DNA traps (extracellular DNA trap cell death (ETosis)). ETosis is a form of cell death, where intracellular DNA is explosively released into the surrounding. It is distinct from apoptosis and necrosis, as DNA remains condensed and compact (86).

## 3 Eosinophils as protective immune cells against filariae

### 3.1 Direct effects of eosinophils against filariae

Eosinophilia is a hallmark of filarial infections and it was shown in humans as well as in mice that eosinophils can contribute to the protective effect against filariae (16). Elevated serum ECP and EDN levels have been observed in different helminth infections, including onchocerciasis, LF as well as schistosomiasis and are indicative of an ongoing infection (87). Furthermore, studies have shown that eosinophils respond in particular to MF and that increased eosinophil counts as well as high levels of eosinophil-derived proteins were associated with the presence of MF in onchocerciasis patients (88). Accordingly, onchocercomata, which are the nodules containing adult *O. volvulus* filariae, contain abundant eosinophils in the presence of MF, while nodules with male worms, dead worms and female worms without MF present significantly lower eosinophil counts. Analysis of onchocercomata with and without MF from the same individual support that this is independent of the host’s immune response (89). In contrast to onchocerciasis patients, in *L. loa*-infected persons, individuals native to endemic regions generally have high MF counts and low eosinophil numbers, while temporary residents show significantly higher eosinophil counts and eosinophil granule protein levels but lower MF counts (90).

Using the *L. sigmodontis* rodent model it was shown that eosinophil numbers increase in the pleural cavity as early as 11 days post infection and thus before adult worms and MF have developed and peak together with the highest MF counts (41, 91). The importance of eosinophils as mediator of protective immunity was demonstrated through several knockout (KO) mouse strains. Indirect evidence for the importance of eosinophils in protective immunity against filariae was obtained by experiments using CCL11/eotaxin-1 and IL-5 KO mice. While IL-5 is the most crucial factor for the development and survival of eosinophils, CCL11 is a chemokine responsible for eosinophil recruitment and activation (51). Even though wild type and eotaxin-1 KO mice had similar larval worm burdens, during patency (adult worms and MF are present) CCL11 KO mice had a significantly higher worm burden in comparison to wild type mice (92). This was accompanied with comparable eosinophil infiltration suggesting that eosinophil recruitment is still mediated in the absence of CCL11. However, CCL11 KO mice harbored eosinophils with reduced activation as shown by CD80 and CD86 expression. Thus, eosinophil activation appears to be crucial for adult worm elimination. Interestingly, only the adult worm burden and not MF numbers were affected by the lack of CCL11 (92). In contrast, mice lacking IL-5 showed reduced eosinophil counts at the site of infection as well as a higher adult worm and MF burden (16). Moreover, these mice presented prolonged microfilaremia and delayed adult worm clearance. Additionally, IL-5 appeared to be crucial for worm containment since IL-5 KO mice presented less nodule formation (93). Accordingly, IL-5 transgenic mice, which overexpress IL-5, exhibited enhanced macrophage and eosinophil attachment to the larvae as early as 10 days post infection. This resulted in increased nodule formation during patency as well as lower adult worm numbers (94). Interestingly, IL-5 overexpression accelerated larval growth during the early infection, suggesting that eosinophil-mediated protection increases developmental pressure on the larvae (94, 95). However, studies have shown that IL-5 signaling has not only an impact on eosinophils but may affect neutrophils as well (96). Thus, protection mediated by IL-5 may not be exclusively caused by eosinophils.

Direct evidence for the importance of eosinophils in mediating protection against filariae was demonstrated in studies using infections in dblGATA mice, which have IL-5 signaling but lack eosinophils. Similar to the IL-5 KO mice, dblGATA mice showed a higher worm and MF burden with an earlier onset of microfilaremia and prolonged maintenance of the infection compared to wild type controls (16). While only 50% of wild type mice became MF positive, all dblGATA mice developed peripheral blood microfilaremia.

Moreover, another study showed that eosinophils control adult *B. malayi* infections. While IL-4Rα KO mice were still able to generate eosinophilia and controlled the infection, mice became susceptible after depleting eosinophils through CCR3. Thus, the adult worm control was apparent even in the absence of IL-4Rα signaling, highlighting the importance of eosinophils and not AAMs in controlling adult worm burden (30).

Thus, IL-5 and eosinophils appear to significantly contribute to the clearance of chronic infections; attacking adult worms and MF.

Filariae are master modulators of the host immune response and there is indirect evidence that filariae also modulate eosinophil-mediated protection against filariae. The release of *Wolbachia* bacteria from adult *O. volvulus* attracts and initiates a strong neutrophil response. Onchocercomata of untreated cattle infected with *O. ochengi* showed mostly neutrophil accumulation, while eosinophils were rarely seen around the adult worms. However, oxytetracycline treatment of *O. ochengi* depleted *Wolbachia* resulting in an increase in eosinophils and eosinophil degranulation around the filariae. This however, required prolonged antifilarial treatment to permanently deplete *Wolbachia* otherwise neutrophils returned to the onchocercomata (97). Thus, antibiotic treatment, leading to the depletion of *Wolbachia* bacteria, replaced neutrophils with eosinophils, which degranulated at the cuticle of the worms (98). On the other hand, direct macrofilaricidal drugs, which did not deplete *Wolbachia*, failed to induce eosinophil accumulation and degranulation (10). Thus, it has been suggested that the release of *Wolbachia* may confer the longevity of the filariae through a defensive mutualism, leading to neutrophil accumulation and preventing effective eosinophil defense (10). This masking may be driven through neutrophil trap formation, which was shown in *O. volvulus* onchocercomata. *Wolbachia* triggered the release of neutrophil DNA traps and thereby prevented eosinophil accumulation (8, 99). Thus, an evolutionary adaptation of the filariae may has developed to reduce eosinophil-mediated protection and increase its survival in the host.

### 3.2 Eosinophil proteins and eosinophil traps against filariae

Protective effects of eosinophils against filariae are mainly mediated through toxic proteins released by the eosinophils. In general, granulocyte-related proteins such as ECP and EDN are increased in patients with onchocerciasis and bancroftian filariasis and the levels of those proteins exceeded by far the levels described for other eosinophil-related diseases, e.g. bronchial asthma and atopic dermatitis. Thus, ECP and EDN serum levels are biomarkers reflecting an ongoing filarial disease (87, 100).

The protective effect of eosinophil granular proteins was shown in mice lacking EPO and MBP, which rendered mice more susceptible to a *L. sigmodontis* infection (74). Shortly before adult worm development, a higher worm burden was recovered from the pleural cavity of these mice compared to control mice. This may be due to the lack of the direct effect of the eosinophil-derived proteins in attacking the filariae, but also indirectly through modulating the subsequent immune response. Lack of MBP for example resulted in increased IL-10 expression in macrophages, while EPO deficiency increased the IL-5 production by CD4+ T cells. Thus, eosinophils and their mediators modulate type 2 immune responses, which affects filarial elimination (74, 101). Deposition of eosinophil proteins on the surface of filariae is a common feature of filariae and direct cytotoxic effects of eosinophil proteins on filariae are supported by several *in vitro* studies. MBP was detected on the surface of *O. volvulus* MF in the skin during the Mazzotti reaction, which is an inflammatory response caused by dying MF following topical administration of DEC in onchocerciasis patients (75). Similarly, ECP and EPO were found on the surface of *O. volvulus* MF in the lymph node after oral treatment with ivermectin (78). MBP deposition was also detected on *B. malayi* MF in a model of TPE (76). *In vitro*, these proteins showed marked anthelminthic activity. MBP, EDN and ECP killed *Brugia pahangi* and *B. malayi* MF in a concentration dependent manner. EDN was the least effective, while EPO, in the presence of ROS, was extremely potent even at low concentrations (77) (**Box 1**).

Another important mechanism of granulocytes is the release of DNA traps. While smaller pathogens such as viruses and bacteria can be phagocytosed, multicellular pathogens like filariae need to be contained through a different mechanism. Especially for neutrophils, DNA trap formation has been shown in various studies as a response to helminths and other pathogens (44, 102–105). Similarly, eosinophils are able to release DNA traps (106). However, even though eosinophils are important players in the defense against helminths, little is known about eosinophil ETosis (EETosis) in response to parasites in general and filariae in particular. So far, EETosis has only been shown in response to the nematodes *Haemonchus contortus* (105) and *Strongyloides ratti* larvae (103). Regarding EETosis in response to filariae, we showed for the first time that eosinophils released DNA traps not only in response to MF of *L. sigmodontis* but also in response to MF of the canine heartworm *Dirofilaria immitis*, suggesting a conserved mechanism. *In vitro* blocking studies demonstrated that the DNA release during EETosis was mediated by the dectin-1 receptor. *In vitro,* the DNA traps entrapped the MF and reduced their motility. *In vivo*, *L. sigmodontis* MF coated with eosinophil DNA traps were cleared faster from the circulation, suggesting that these traps may be an essential mechanism in MF clearance (102).

Of note, for human eosinophils it was shown that CLCs form during the process of EETosis. As murine eosinophils lack galectin-10, the protein responsible for CLC formation (79), additional studies using human eosinophils have to be performed to elucidate the specific role of CLCs in EETosis and their effect on filariae.

### 3.3 Eosinophils and vaccine efficacy against filariae

Next to mass drug administrations and the development of new drug candidates (2), current attempts to eliminate filarial diseases include the development of a potent anti-filarial vaccine, which would help to control the transmission of the disease (107). Several animal studies investigated the protective effect using irradiation-attenuated L3 larvae (irr. L3), which results in protective effects ranging from 30 to 100% (108–113). The immune reaction towards the irr. L3 appears to be dependent on larval-specific antibodies as well as eosinophils. In jirds vaccinated with irr. *Acanthocheilonema viteae-* or *B. malayi* L3, eosinophil-rich granuloma were observed after challenge infection and strong eosinophil degranulation was associated with the destruction of the worm cuticles (114, 115). *O. volvulus*-vaccinated and subsequently challenged mice were also able to kill the in diffusion chambers intraperitoneally implanted adult filariae. Eosinophils were the only cells that accumulated in the diffusion chambers of the immunized mice and depletion of IL-5 prevented the protective effect (29). Similarly, vaccination efficacy in *L. sigmodontis*-infected mice was dependent on eosinophils and IL-5. After challenge infection of mice vaccinated with irr. L3, IL-5 and eosinophils accumulated in the skin and incoming L3 larvae were eliminated within the first 2 days post infection (82, 83). In accordance, anti-IL-5 antibody treatment, suppressed tissue eosinophilia and prevented the protection (82).

Vaccination-mediated protection was also dependent on B cells and antibodies. Lack of B cells and antibodies in µMT mice abolished the protective effect even though eosinophil infiltration occurred at the site of infection (116). In this context, degranulation of eosinophils was significantly reduced in immunized and challenged µMT mice compared to wild type mice, suggesting that antibody-complexes are required for eosinophil effector function through ADCC (116). In accordance, *in vitro* motility inhibition of intestinal nematode *S. ratti* L3 larvae by eosinophils was dependent on antibodies from infected animals. Only in the presence of antibody-containing plasma from infected but not naïve animals, *S. ratti* L3-induced DNA release by eosinophils inhibited the larval motility (103).

**Figure 2:**
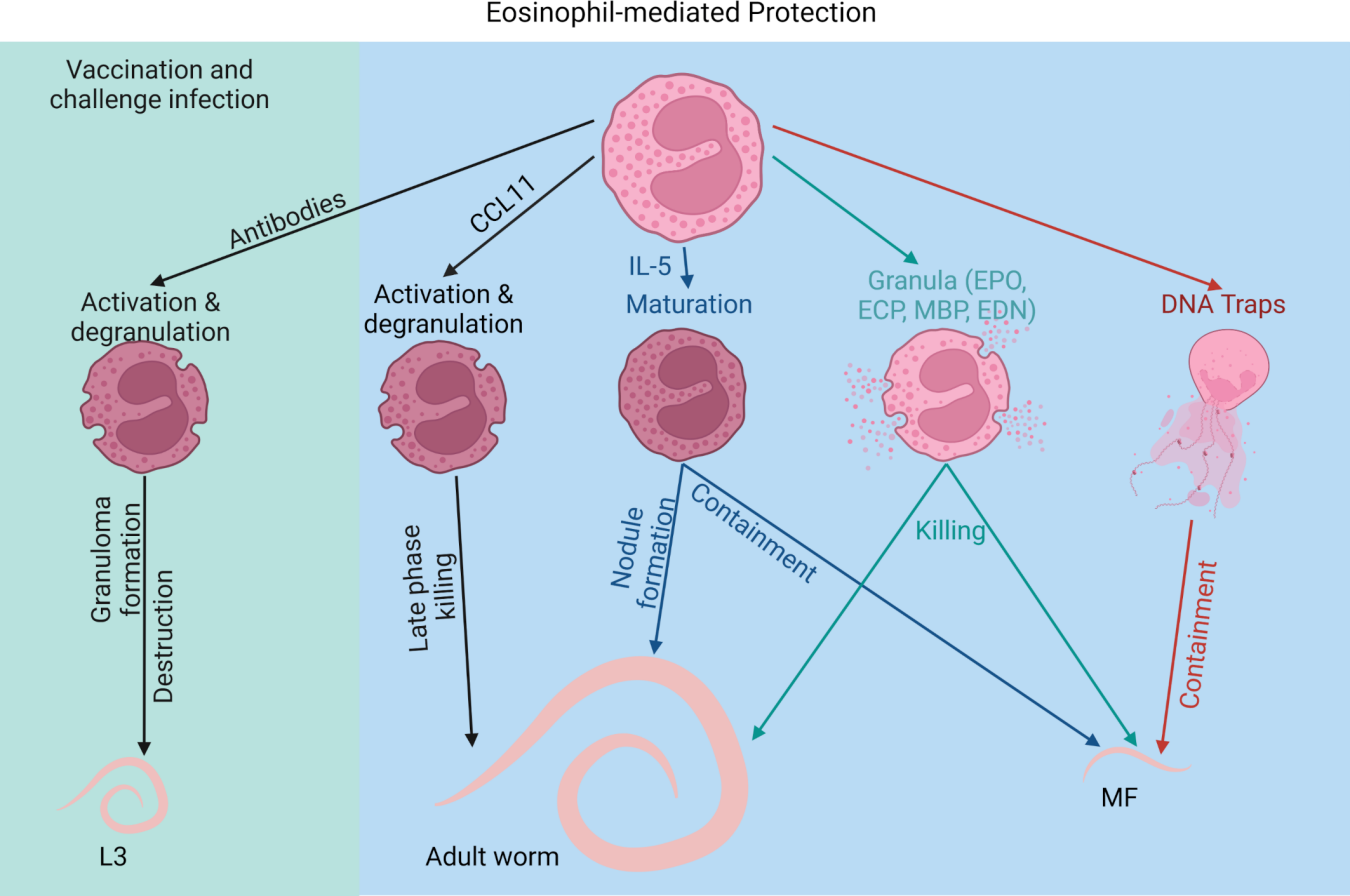
Eosinophil-mediated protection against filarial infections. Created with BioRender.com.

In summary, eosinophils not only contribute to protection during primary infection in response to adult worms and MF, but are crucial for mediating protection during vaccinations (**Figure 2**).

## 4 Eosinophils and filarial pathology

By successfully inducing an immunosuppressive milieu, filariae hamper protective immunity and prevent at the same time the development of pathology that, in filariasis patients, is a result of an unbalanced immune response towards the filariae (117). Thus, a balanced host response includes sufficient parasite control and maintenance of immune homeostasis without excessive tissue damage. Failure of this balance results in excessive immune-mediated inflammation and thus, in pathology. Clinical manifestations observed in the different filarial diseases are a result of inflammation towards different filarial stages. While pathology in LF patients (lymphedema, hydrocele and elephantiasis) and Calabar swelling in loiasis patients is a result of the immune reaction to the adult worms, dermatitis and ocular lesions in onchocerciasis patients and TPE in LF is caused by the response to dead MF.

### 4.1 Eosinophils and pathology: Adult worms

Lymphedema, hydrocele and elephantiasis in LF patients is primarily a result of excessive immune responses towards the adult worms residing in the lymphatic vessels. However, the impact of eosinophils on pathology development is less well understood. The pathology development is primarily due to a failure to induce T cell hyporesponsiveness (118). Vascular endothelial growth factor released by endothelial cells and the secretion of pro-inflammatory cytokines have been implicated in LF pathology by enhancing leucocyte adhesion, increasing vascular permeability and promoting lymphangiogenesis. Increased frequencies of Th1, Th9 and Th17 cells and reduced numbers of Th2 cells are associated with filarial pathology (118, 119). In addition, elevated levels of innate pro-inflammatory cytokines such as C-reactive protein, IL-6, IL-8 and TNF in the peripheral blood have been associated with disease pathology (118), while only indirect evidence for the contribution of eosinophils exist. Locally, dysregulation of matrix metalloproteinases (MMPs) and their inhibitors (TIMPs) (120), which are released by a variety of cells including macrophages, epidermal cells, fibroblasts and granulocytes, are frequently linked to filarial pathology as well as elevated levels of type 2 cytokines, e.g., IL-5, IL-13 and TGFβ (121). This was further studied using a *B. malayi* infection model in ferrets. There, infective L3 larvae were injected into the hind-footpad of ferrets inducing LF pathology, including parasitological, immunological and histological changes remarkably similar to human filarial infections. Using this model, it was shown that perivascular and subcutaneous inflammation was associated with increased infiltration of plasma cells and histiocytes and also with increased neutrophil and eosinophil numbers (28). Thus, eosinophils and eosinophil-associated IL-5 may be involved during LF pathology development. However, in *W. bancrofti* patients, the pathology was rather connected to decreased serum fibrosis markers and increased levels of hyaluronan, while serum ECP and EPO concentrations were not altered among patients with lymphatic pathology compared to asymptomatic patients. An increase in ECP levels at the limit of significance was observed in patients with elephantiasis (100).

An additional example of the importance of unregulated immune responses is seen in loiasis pathology development. Temporary residents, with less exposure to the parasites, show a significantly higher prevalence for Calabar swellings compared to persons native to the region. Calabar swellings are edema in the subcutaneous tissue, caused by the migrating adult filariae. The prevalence of Calabar swellings in temporary residents was associated with elevated levels of eosinophil numbers, eosinophil-associated proteins IL-5 and GM-CSF, as well as filarial-specific IgG. On the other hand people native to endemic countries showed immune tolerance with reduced eosinophil numbers and eosinophil-associated responses, higher MF loads and lower frequencies of pathology (90). Thus, eosinophil presence and responses are related to disease pathology in loiasis patients.

In summary, while many animal studies underline the importance of eosinophil-mediated protection against adult worms, evidence for eosinophils contributing to pathology based on inflammatory responses towards the adult worms is only indirect.

### 4.2 Eosinophils and pathology: Microfilariae

#### 4.2.1 Lung pathology and tropical pulmonary eosinophilia (TPE)

A severe pathology observed in some LF patients is TPE caused by MF of *W. bancrofti* and *B. malayi*. It is characterized by increased peripheral eosinophil numbers and asthma-like symptoms, i.e., cough, dyspnoea and wheezing. The symptoms result from an immunological hyperresponsiveness to the MF and patients show high levels of serum IgE and filarial-specific antibodies (11, 122, 123). Thereby, MF are trapped and cleared in the pulmonary circulation. As a result, microfilaremia is rarely observed in TPE patients. MF trapping leads to the release of antigens triggering chronic inflammation with peribronchial and perivascular exudates as well as acute eosinophil infiltration. Within the lower airway, severe eosinophilic inflammation occurs and eosinophils massively release their granules into the lung parenchyma. EDN has been found to be increased in bronchoalveolar lavage (BAL) of patients with TPE. MBP-2, which is only found in eosinophils, is also increased and may be a suitable biomarker for TPE. Especially MBP has been associated with airway hyperreactivity leading to fibrosis. Thus, eosinophils and their granules serve a dual role by eliminating MF and by inducing lung pathology (11, 124).

Indirect evidence that eosinophils are responsible for TPE pathology is given by studies demonstrating that individuals treated with DEC, which removes MF, leads to a marked decrease in lung eosinophilia and an improvement of the clinical symptoms (125). Patients treated with DEC continue to show a mild persistent inflammation with sustained eosinophilic alveolitis as well as higher free oxygen radicals in the bronchoalveolar fluid (126). Thus, the immune response by eosinophils towards the MF rather than the MF themselves are responsible for the immunopathology.

Direct evidence of eosinophil-mediated lung damage during TPE was demonstrated in a TPE-mouse model, where freeze-thawed *B. malayi* MF are used to sensitize mice followed by a challenge with viable MF (127). Neutralization of the α4 and β7 integrins, which mediates eosinophil migration, prevented pathology development, reduced peripheral eosinophilia, reduced lung eosinophil infiltration, and subsequently minimized lung damage (32). However, it has to be mentioned that neutralization of α4 and β7 integrin also impairs the migration of several T helper cell subsets to the lung. Thus, the results may not exclusively demonstrate the impact of eosinophils on lung damage.

Skewing the type 2 immune response towards a more pronounced type 1 response by administering IL-12 significantly reduced IgG1 and IgE levels, eosinophilia as well as MBP levels and lessened lung pathology in the TPE mouse model (76). Further evidence for eosinophil-mediated pathology during TPE was shown in mice lacking IL-5, which prevented peripheral and pulmonary eosinophilia as well as airway hyperresponsiveness (128). Similarly, our own unpublished results show that TPE induction using *L. sigmodontis* MF in dblGATA mice, which lack eosinophils, resulted in reduced lung pathology.

Even in asymptomatic LF patients, eosinophils have been shown to induce clinical symptoms after treatment. Following DEC treatment, patients often develop symptoms such as fever, headache and lethargy and this is accompanied by a significant increase in IL-5 levels followed by an increase in eosinophil counts (129, 130). One study conducted by Gopinath et al. closely followed the cytokine and chemokine response as well as eosinophil blood counts after treatment (129). Four hours following treatment, patients developed clinical symptoms, which peaked 24h after treatment. This was accompanied by a drop in peripheral eosinophil counts reflecting the immediate migration of eosinophils into the tissue or adhesion to dying MF followed by a significant eosinophil increase within 3-4 days post treatment. The eosinophil-mediated response was also reflected in the increase in eosinophil-associated cytokines and chemokines such as IL-5 and CCL5/RANTES as well as the increase in the eosinophil-associated proteins MBP and EDN in the peripheral blood upon treatment. Moreover, DEC treatment increased the expression of the integrin markers VLA-4, α4 and β7, which mediate eosinophil adhesion to endothelia or to dying MF (129).

During natural infection with *L. sigmodontis*, eosinophil-mediated lung pathology is also observed. Adult *L. sigmodontis* worms, residing in the pleural cavity, release MF that are thought to enter the peripheral blood *via* the lung capillaries, inducing lung injury. It was shown that MF in *L. sigmodontis*-infected jirds induced fibrotic polyps on the visceral mesothelium with high infiltrates of macrophages, lymphocytes and eosinophils (131). In mice, hypertrophic mesothelium was seen in infected wild type as well as in eosinophil-deficient mice regardless of the presence of MF. However, hyperplasia was mainly seen in MF-positive wild type mice and was absent in MF-negative wild type mice and mice lacking eosinophils (131). Therefore, eosinophils not only contribute to protective immunity against MF but also to pathology development during LF and murine filariasis.

#### 4.2.2 Skin and eye pathology

Pathology in onchocerciasis patients is induced by the immune response towards the dying MF in the skin leading to dermatitis with the most severe form of chronic hyperreactive onchocerciasis called sowda, and in the cornea leading to vision impairment and even blindness (132). Most individuals living in endemic areas have acquired a parasite-specific anergy with reduced cellular responses and modulated inflammatory responses. Hyperreactive response to the parasite antigen on the other hand results in pathology, which is associated with high parasite-specific IgE and IgG levels as well as absence of skin MF (133). Moreover, in ocular lesions, MF migration itself is not responsible for eliciting pathology, but clinical manifestations result from the inflammation in response to the dead/dying MF; in the eye this leads to antigen release that triggers a local inflammatory response, resulting in corneal inflammation characterized by an opaque area called punctate keratitis. Heavily infected individuals with high MF numbers in the cornea develop sclerosing keratitis with scaring of the cornea and in visual impairment (134).

To study the immune response in the cornea of onchocerciasis patients, an *O. volvulus* keratitis mouse model helped to decipher the mechanism of immunopathology. To induce ocular lesions, mice are treated with intraconjunctival or intrastromal injection of viable MF, or with intrastromal injections of *O. volvulus* antigen after immunization with filarial antigen. This drives a Th2 response with local production of IL-4 and IL-5 (26). As a response, a biphasic accumulation of granulocytes occurs, with neutrophils being the first cells to arrive at the site of inflammation followed by eosinophils, inducing inflammation in the cornea leading to ocular lesions (25, 26). So far, it is known that CD4+ T cells and B cells play an essential role during the development of keratitis by driving granulocyte recruitment. Depletion of CD4+ T cells reduced pathology, which was associated with significantly reduced recruitment of eosinophils (135). Similarly, B cell-deficient mice failed to develop corneal disease through impaired recruitment of eosinophils and neutrophils, which was mediated through circulating immune complexes (136). Thus, the presence of eosinophils and neutrophils is associated with disease development. However, the exact mechanism and the contribution of both cell types to the development of keratitis is less well understood. It is assumed that eosinophils and neutrophils both contribute to the inflammation. Thereby, their granular content, which is released into the cornea, is suggested to mediate pathology. For example, *in vitro* it was shown that MBP is highly toxic to corneal cells (137). However, the essential role of eosinophils and neutrophils on disease outcome has still to be demonstrated. In this regard, type 2 and type 1 cytokines modulated severity of keratitis, which was associated with eosinophil numbers. IL-4 KO mice presented reduced keratitis that correlated with impaired eosinophil recruitment to the cornea (138), while IL-12-treated mice experienced enhanced eosinophil recruitment and excessive pathology (139). Thus, eosinophil presence appears to be essential for pathology development. However, IL-5 KO mice develop keratitis in the absence of eosinophils. The biphasic response of neutrophils followed by eosinophils is ablated in these mice resulting in a constitutive presence of neutrophils in the cornea at early and late time points (140). These data suggest, that neutrophils rather than eosinophils mediate corneal opacification. Moreover, neutrophil- but not eosinophil-mediated keratitis was demonstrated in response to *Wolbachia*. Injection of *Wolbachia*-containing worm extract into the cornea of mice induced keratitis, while injection of *Wolbachia*-depleted extract and extracts from the rodent filaria *A. viteae*, which lack *Wolbachia* bacteria, resulted in reduced ocular lesions with lower neutrophil recruitment. While neutrophil recruitment was ablated in the absence of *Wolbachia*, eosinophil accumulation was independent of *Wolbachia* (39). Thus, neutrophils seem to be pivotal for the induction of ocular lesions in onchocerciasis patients, while the exact contribution of eosinophils has still to be determined.

Interestingly, during experimental-induced dermatitis of onchocerciasis, eosinophils and neutrophils follow a similar recruitment pattern as in the cornea. Injection of *O. volvulus* antigen into the ear of immunized mice resulted in dermatitis with intense infiltration of neutrophils followed by eosinophils (141). Thus, a similar mechanism during skin manifestations and keratitis may be in place. However, while the role of eosinophils in ocular lesion may be questionable, a more prominent role of eosinophils during onchocerciasis-related skin manifestations has been observed. Sowda patients show significantly higher serum levels of ECP in comparison to hyporesponsive generalized onchocerciasis patients. Especially MF density is low or absent in sowda patients; thus, increased ECP levels could reflect a higher degree of eosinophil activation and MF killing (87).

Since dying MF elucidate an eosinophil-mediated response, pathology is observed after DEC treatment in onchocerciasis patients due to the rapid death of MF in the skin and the cornea. It was already described in 1984 that onchocerciasis patients treated with DEC showed marked inflammation in the skin characterized by extensive eosinophil infiltration into the dermis and epidermis with intradermal abscesses containing dead MF and eosinophils (75), which was mediated through eotaxin release (60). Interestingly, only dead and dying MF were coated by MBP, while viable MF did not stain for MBP suggesting that eosinophils damage the MF of *O. volvulus* through the release of toxic proteins such as MBP (75). Similarly, dead MF elucidated higher DNA release by eosinophils in comparison to viable MF *in vitro* (102). The eosinophil-associated inflammation in the skin of DEC-treated onchocerciasis patients is referred to as Mazzotti reaction. After oral treatment with DEC, peripheral eosinophil counts first decrease followed by a significant increase in peripheral blood eosinophil counts as well as an increase in eosinophil-associated proteins (EDN, MBP), which coincide with the infiltration and degranulation of eosinophils in the papillary dermis (142). In addition, neutrophils and their proteins are found around the dying MF in the skin and probably work together with the eosinophils to kill MF after DEC treatment (143). Therefore, onchocerciasis patients should not be treated with DEC to prevent the strong immune reaction towards the MF and thus development of ophthalmological and dermal pathology (2).

Similarly, ivermectin treatment in onchocerciasis patients induced a significant increase in CCL5/RANTES and eotaxin expression in the skin post treatment, which resulted in an increase of skin eosinophils 24h after treatment (144). Moreover, eosinophils, together with macrophages, mast cells and some T cells, encircled degenerated MF after ivermectin treatment of hyperreactive onchocerciasis patients (145). Skin MF were markedly reduced after treatment and primarily found in the regional lymph nodes, where they were surrounded by eosinophils and eosinophil-derived toxic deposits such as ECP, EPO and cationic leukocyte antigen (CLA) (78, 146, 147). This clearance of MF within lymph nodes of ivermectin-treated patients renders this treatment safe for use of onchocerciasis patients, preventing the risk of permanent visual impairment.

DEC or ivermectin treatment, which causes a rapid decline in MF, can also result in post-treatment adverse events in loiasis patients with high MF counts. For DEC treatment clinical manifestations were associated with eosinophil infiltration into the skin and subsequent migration to peripheral lymph nodes (148). A study investigating loiasis in temporary residents showed that DEC treatment resulted in severe adverse events in 7 out of 20 patients with endomyocardial fibrosis and renal disease, which was associated with increased eosinophil numbers, IgE and filarial-specific antibodies (149). On the other hand, treatment of loiasis patients with reslizumab, an anti-IL-5 antibody, decreased absolute eosinophil counts in the peripheral blood, but had no effect on MF clearance nor did it lead to an improvement of adverse events suggesting that eosinophil depletion has less impact on post-DEC adverse events in loiasis patients than assumed (150).

**Figure 3:**
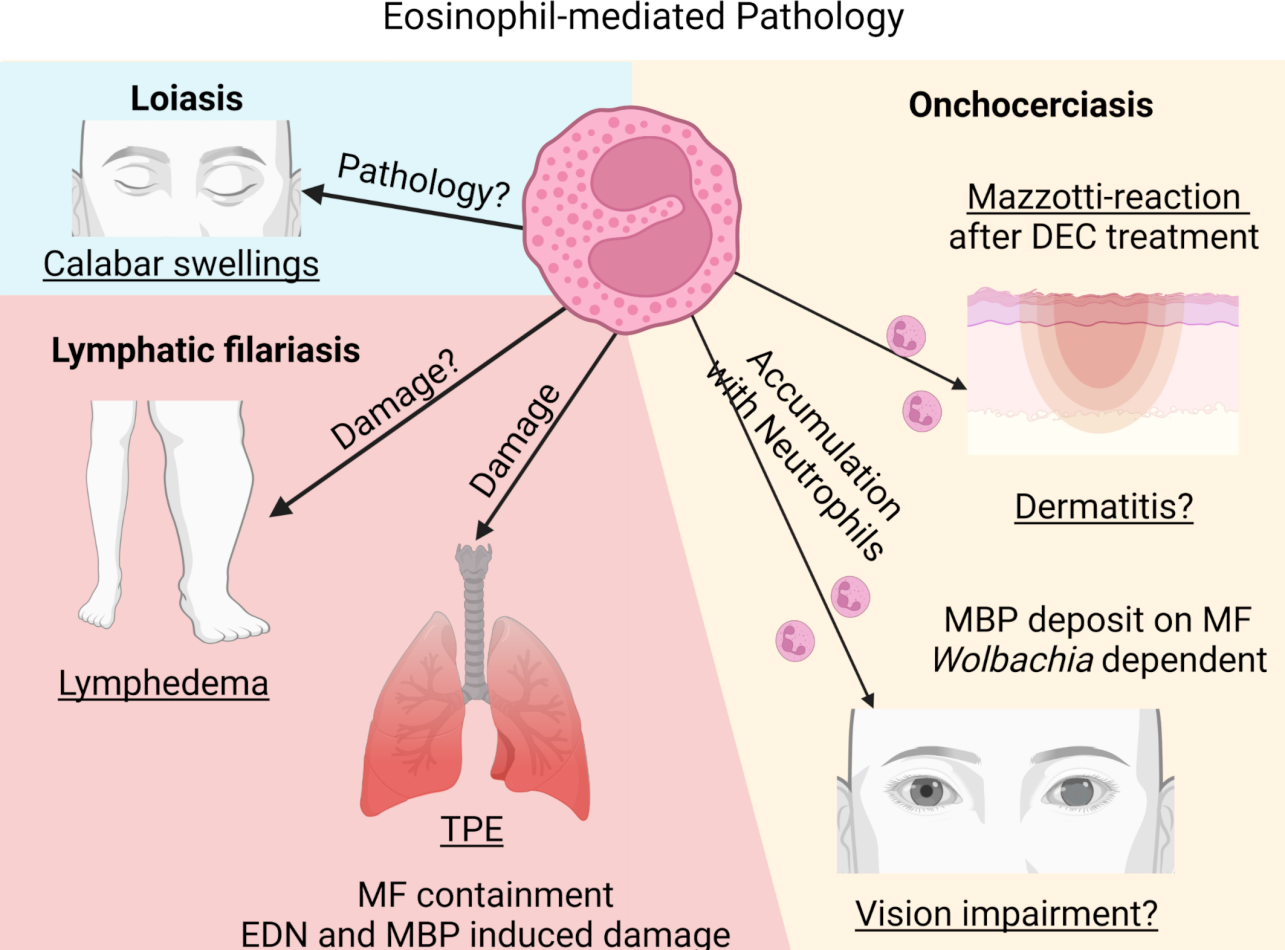
Eosinophil-mediated pathology during filarial infections. Created with BioRender.com.

These data indicate that eosinophils may contribute to skin and eye pathology in onchocerciasis patients. Especially after treatment with DEC, eosinophil responses coincided with the clinical symptoms in onchocerciasis patients. Based on the data received from animal experiments, it can be assumed that eosinophils promote MF killing and may support microfilaricidal drug action (**Figure 3**). However, some of the studies are contradictory for the role of eosinophils and further research is required to define the specific role of eosinophils and neutrophils in the development of filarial pathology.

## 5 Gaps in understanding and future work

Eosinophils serve several roles during filarial infection by promoting protection on the one hand and contributing to pathology development on the other hand. The great task protective immunity has to achieve is a balanced host response, which includes sufficient parasite control and maintenance of immune homeostasis without excessive tissue damage.

Even though eosinophil function during protective immunity and disease pathology in filarial infection has been studied, several questions remain unanswered.

### 5.1 Direct effects of eosinophils on protection

While granular proteins appear to be involved in protective immunity against filariae, the role of DNA traps in filarial containment has still to be elucidated. While smaller pathogens can be phagocytosed, larger multicellular organisms such as filariae need to be contained through a different mechanism. Thus, it can be assumed that this may be mediated through extracellular DNA traps to prevent parasite escape and to concentrate and focus granular proteins on the surface of the parasite and thus limit excessive systemic inflammation. Such a response was observed for the fungi *Aspergillus fumigatus* that was entrapped in the lung by neutrophil DNA traps that contributed to fungal killing (151). Since eosinophils can inhibit MF motility *in vitro* and DNA traps support MF removal from the peripheral circulation (102), the question remains where those entrapped MF are removed (role of spleen, lung and other organs), if eosinophils also contribute to the containment of MF after their release from the adult worms, and if ETosis supports drug-induced MF removal. Furthermore, with Dectin-1 the receptor inducing EETosis was identified (102), however, the structure on the MF that induces EETosis is not yet known. Given that dead MF lead to a stronger induction of EETosis, PAMPs and DAMPs from dead and viable MF may differ or use different pathways.

Moreover, the role of complement-mediated toxicity and ADCC for eosinophils during primary filarial infection is not completely known. Antibodies and ADCC appear to be an important mechanism during secondary infections and after vaccination, however, during primary infection little is known. It is known that IgG4 from MF+, MF- LF patients and endemic normal but not from LF patients with pathology can inhibit the activation of granulocytes, suggesting that antibody-mediated toxicity by granulocytes may contribute to pathology in LF patients (152). However, the contribution of ADCC by eosinophils for protection against filariae is not known. For other helminth infections, we know that antibodies and complement contribute only marginally to the protective effect by eosinophils during primary infection. Eosinophil-mediated immunity against *Nippostrongylus brasiliensis* is independent of complement (153) and antibody-dependent killing of *Schistosoma mansoni* requires additional eosinophil activation or pre-exposure of the eosinophils to the parasite (154). Similarly, *S. ratti* L3 larvae motility was only inhibited by immune serum and not in the presence of naïve serum suggesting that helminth-specific antibodies are required to attack the worm (103). However, the exact role of complement and antibodies during primary filarial infection needs to be elucidated yet.

### 5.2 Interaction of eosinophils with other immune cells during filarial infections

Depletion experiments and usage of KO mice suggest specific roles of eosinophils and neutrophils during filarial infection, however kinetics on granulocyte recruitment indicate a close interplay of eosinophils and neutrophils during protective immunity as well as pathology development (155). However, the exact interplay between eosinophils and neutrophils during filarial infection remains unclear. This includes the recruitment and activation of each other, their interaction during the containment and elimination of the filariae, as well as the development of pathology, e.g. ocular lesions and skin manifestations in onchocerciasis patients. The current results suggest that eosinophils are recruited after neutrophil accumulation in the skin and cornea of onchocerciasis patients. The question remains if neutrophils directly induce eosinophil recruitment or if this is mediated through T cells. Further, it has to be clarified which cell type is responsible for pathology development in onchocerciasis patients and if both granulocyte populations have redundant roles.

Moreover, eosinophils seem to interact with macrophages during filarial infection (30, 96), but more experiments analyzing the exact mechanism on how eosinophils impact macrophage plasticity locally and systemically during filarial infection are required.

In addition, in diseases such as allergies (156), eosinophils affect T cell responses by promoting T cell proliferation, activation and polarization through type 1 and type 2 cytokine release (157). Such an unbalanced T cell hyperresponsiveness was also associated with the occurrence of lymphedema in LF patients, but the exact role of eosinophils in this context is not known. The interaction of eosinophils and T cells was suggested in patients suffering of two eosinophil-driven diseases, allergy and filarial infection. Concomitant filarial infection and allergy increased parasite antigen-driven Th2, Th9 and regulatory T cell-related cytokine expression in comparison to filaria-infected and non-allergic patients. This correlated with IgE levels, eosinophil numbers and their degranulation products (158). The authors suggested that this particular immune response could affect parasite control and associated pathology.

### 5.3 Direct effects of eosinophils on pathology

While the role of eosinophils in hyper- and hyporesponsive onchocerciasis patients has been shown, the causative effect remains unclear. Hyperresponsive patients present marked eosinophil responses, while hyporesponsive patients are characterized by reduced eosinophil numbers and responses. Thus, the differences in immune status among patients appear to be a result of an unbalanced immune responses towards a more pronounced Th17 and Th1 shift in hyperreactive onchocerciasis patients rather than a regulatory response in hyporesponsive patients. Eosinophils are able to produce type 1 and type 2 cytokines and different subtypes of eosinophils have been described including resident eosinophils that rather show an anti-inflammatory phenotype, responsible for tissue homeostasis, and inducible eosinophils with a marked pro-inflammatory phenotype (159). Thus, eosinophils and their different phenotypes could contribute to the different disease outcomes among onchocerciasis patients and explain why some patients develop pathology, while others remain asymptomatic and maintain a high parasite burden. Differences in the composition of resident and inducible eosinophils could be also the case for patients suffering from TPE. Similarly, it can be hypothesized that differences in the ETosis capacity may contribute to disease pathology, which, when balanced, limits inflammation and clears filariae, while excessive ETosis may drive pathology. Thus, future studies should analyze the composition of eosinophil phenotypes in the different filarial disease settings and determine whether filarial immunomodulation is involved.

Even though eosinophil function during the Mazzotti reaction in onchocerciasis patients has been investigated, several questions on the eosinophil-mediated pathology remain unanswered. The exact interplay of eosinophils and DEC has not be elucidated yet and the question remains how and whether the drug impacts eosinophil function directly. Identifying the exact mechanism on drug and eosinophil-responses may open up new and improved treatment strategies for filariasis, which will not only include macro- or microfilaricidal activity but also lessen associated adverse events.

### 5.4 Targeting eosinophils as treatment option

Monoclonal antibodies targeting eosinophil-development are used for other eosinophil-associated pathologies, such as allergic asthma. Mepolizumab and reslizumab, monoclonal anti-IL-5 antibodies, and benralizumab, which targets IL-5Rα, are approved for the treatment of asthma and have shown to improve allergic asthma (160, 161). Thus, similar treatments in filariasis patients could lessen symptoms. However, one study using reslizumab prior DEC treatment in loiasis patients failed to reduce adverse events (150). Moreover, monoclonal antibody treatment for the treatment of filarial pathology faces several limitations. The therapy is extremely cost intensive, requires intense patient care and often requires parental administration. However, most countries affected by filarial infections lack an advanced health care system making monoclonal antibody therapies impossible. Furthermore, drugs currently used for mass drug administration do not kill the adult filariae. Thus, impairing protective immune responses by eosinophils, may facilitate filarial survival and transmission of the diseases. The latter does not only refer to filarial diseases, but also other helminth infections, such as intestinal helminths (162).

## 6 Summary

Eosinophilia and increases in eosinophil protein levels are often associated with an ongoing helminth infection and can serve as first diagnostic indication. Eosinophils provide essential effector functions to control filarial infections but also induce filarial pathology. **Tables 3**, **4** summaries the findings on eosinophil-mediated protection and pathology in human filariasis and in filarial animal models.

**Table 3:** Summary of eosinophil involvement in protective immune responses during filarial infections.

**Onchocerciasis**	General	Humans: • Abundant eosinophils in nodules with MF • Increased ECP and EDN levels in serum
	Adult	Animal model: • *Wolbachia*-depletion: replacement of neutrophils with eosinophils and eosinophil degranulation
	MF	Humans: • DEC treatment: eosinophil proteins on MF
**Lymphatic filariasis**	General	Humans: • Increased ECP and EDN levels in serum
	Adult	No clear evidence
	MF	Animal model: • MBP, EDN, ECP, EPO kill *B. pahangi* and *B. malayi* MF *in vitro* • IL-4Rα KO mice *B. malayi* MF iv: eosinophilia and worm clearance • Treated with anti-CCR3 antibody: susceptible
**Loiasis**		Humans: • Negative correlation of eosinophils/eosinophil products and MF
***Litomosoides sigmodontis***		• Eosinophilia develops bevor patency • Eotaxin-1 KO mice: higher adult worm burden • Same eosinophil counts, reduced eosinophil activation • IL-5 KO, IL-5 transgenic + dblGATA mice: eosinophilia associated with adult worm and MF clearance and nodule formation • MBP + EPO KO mice: increased adult worm burden • Traps contribute to MF clearance *in vivo*

**Table 4:** Summary of eosinophil involvement in pathology development during filarial infections.

**Onchocerciasis**	General	
	Adult	No clear evidence
	MF	Dermatitis: Humans: • DEC/ivermectin treatment: • Increased eosinophil counts + eosinophil-associated protein levels • Dead MF encircled by eosinophils + coated by eosinophil proteins Animal model: neutrophil and eosinophil accumulation
		Ocular lesions: Animal model: • Neutrophil + eosinophil accumulation, responsibility of cell types not completely determined • Eosinophil contribution: IL-4 KO mice + IL-12-treated mice: correlation with eosinophil number and inflammation • Neutrophil contribution: IL-5 KO mice + *Wolbachia*-injected mice: keratitis and no eosinophil but neutrophil accumulation
**Lymphatic filariasis**	General	
	Adult	Lymphedema: Humans: Increased IL-5 Animal model: Increased eosinophil numbers
	MF	TPE: Humans: • Acute eosinophil infiltration and eosinophilic inflammation • Eosinophil-associated proteins in BAL and serum • DEC treatment: reduced MF counts and eosinophil inflammation Animal model: • Neutralization of α4 and β7 integrins, IL-12 administration, IL-5 KO mice + dblGATA mice: reduced lung damage and reduced eosinophilia
**Loiasis**		Calabar swelling: Humans: • Low MF loads associated with increased eosinophil numbers and IL-5 • DEC treatment: endomyocardial fibrosis + renal disease associated with eosinophils • Anti-IL-5 treatment: no impact on MF clearance and SAEs
***Litomosoides sigmodontis***		Infection: hyperplasia lung pathology during patency • Mainly in MF-positive animals, absence in dblGATA mice

Although a direct evidence for the role of eosinophils is not always given, animal experiments convincingly demonstrate that eosinophils are important to control microfilaremia and adult worm burden. Moreover, eosinophils are shown to be of particular importance for vaccination efficacy in animal models. With regard to filarial pathology, eosinophil-mediated inflammatory responses against MF are likely supporting the development of filarial pathology, as discussed for the development of keratitis and dermatitis in onchocerciasis patients or lung inflammation during TPE in LF patients. Similarly, following DEC treatment, eosinophil- rather than neutrophil-mediated responses seem to trigger skin lesions in onchocerciasis patients. Future studies will have to further elucidate the exact role of the different eosinophil phenotypes during filarial infection and distinguish them from the role of neutrophils. Further research on eosinophil function, eosinophil plasticity and interaction with other immune cells is required.

## Author contributions

Writing original draft: AE and MH. Writing review and editing: AE, AH, and MH. All authors contributed to the article and approved the submitted version.

## Funding

MH and AE are funded by the German Center for Infection Research (DZIF Translational Thematic Unit: Novel Antibiotics grant #09.701) and by the Deutsche Forschungsgesellschaft (DFG grant HU2144/3-1). AH received support from the DZIF (#09.807, #09.806, #09.822 and #09.914), the Deutsche Forschungsgesellschaft (DFG grant PF673/3-1) and the Federal Ministry of Education and Research (BMBF, grant 16GW0227K).

## Conflict of interest

The authors declare that the research was conducted in the absence of any commercial or financial relationships that could be construed as a potential conflict of interest.

## Publisher’s note

All claims expressed in this article are solely those of the authors and do not necessarily represent those of their affiliated organizations, or those of the publisher, the editors and the reviewers. Any product that may be evaluated in this article, or claim that may be made by its manufacturer, is not guaranteed or endorsed by the publisher.
